# Tackling aging and chronic disease: How space exploration and the ‘chameleon effect’ of pharmaceuticals can lead the way

**DOI:** 10.1113/EP092652

**Published:** 2026-08-18

**Authors:** Carina Kern, Tina Woods, Keith Siew

**Affiliations:** ^1^ LinkGevity, Babraham Research Campus Cambridge UK; ^2^ International Institute of Longevity (IIOL) Warsaw Poland; ^3^ Collider Health UK; ^4^ London Tubular Centre Royal Free Hospital London UK; ^5^ Centre for Kidney and Bladder Health University College London London UK

**Keywords:** aging, chameleon effect, chronic disease, kidney disease, loss of resilience, necrosis, pharmaceuticals, space biology, therapeutics

## Abstract

A common misconception surrounding space exploration is that humanity's pursuit of interplanetary travel, such as missions to Mars, is an escape from Earth. In reality, this endeavour offers an invaluable opportunity to address critical biomedical challenges here at home. The extreme conditions of space, including microgravity, cosmic radiation and prolonged isolation, create a natural laboratory for studying human biology under stress, allowing us to explore the boundaries of human resilience. These exposures yield key insights into the biology of aging, the accelerated decline of physiological function and the early onset of chronic diseases. Among the organs most susceptible to environmental stress is the kidney, which is at high risk of failure during long‐duration spaceflight. Its sensitivity makes it an ideal model for understanding how stress accelerates degeneration, both in space and on Earth. By studying kidney function alongside broader cellular processes, we can illuminate how stress drives aging and chronic disease. This is particularly exemplified by the process of cellular necrosis (unprogrammed cell death), which represents a key central node as recently demonstrated. Necrosis acts upstream of processes like cellular senescence and tissue degeneration, and is the mechanism through which multiple environmental factors erode resilience and drive the progression of chronic diseases like kidney disease. Following this exploration of how stress accelerates biological decline, we examine strategies that may protect against such damage, preventing disease and preserving physiological resilience. Pharmaceuticals, with their ability to modulate physiology transiently, are especially powerful in dynamic, real‐world environments where stressors continually change. This ‘chameleon effect’ of pharmaceuticals offers the potential to uncouple biological trade‐offs and enhance adaptive capacity to stress. Understanding these processes in the space environment provides an unprecedented opportunity, difficult to reproduce on Earth, to identify links between exposure and develop targeted interventions to enhance health and resilience on Earth.

## Abstract (highlights)

- **What is the topic of this review?**
  The paper explores how space exploration and spaceflight‐related stressors can be used to better understand aging and chronic illness, while examining how pharmaceuticals may enhance human resilience to these stresses.
- **What advances does it highlight?**
  It highlights new insights into how environmental stress accelerates aging, cellular damage and chronic disease, particularly through cellular necrosis; and introduces the ‘chameleon effect’ of pharmaceuticals as a dynamic strategy to enhance resilience and enable targeted therapies for aging and chronic diseases on Earth and during space exploration.

## 1 NECROSIS VERSUS PROGRAMMED CELL DEATH

Cells are the core building blocks of life. Their death can be either a carefully orchestrated process to maintain survival (programmed cell death) or the defining driver of biological degeneration (necrosis). Necrosis is distinct from programmed forms of cell death, such as apoptosis, autophagy, necroptosis and ferroptosis, which are genetically regulated and play adaptive roles in maintaining tissue homeostasis and preventing diseases such as cancer. Programmed cell death typically involves autonomous cellular dismantling that avoids eliciting inflammation. In contrast, necrosis is a destructive, unregulated (not adaptive) process triggered by overwhelming stress, i.e., intrinsic or extrinsic perturbations (Buja, 2021; Kern et al., 2025; Khalid & Azimpouran, 2024). It results in the rupture of cellular membranes, releasing toxic intracellular contents that provoke inflammation and tissue damage. Necrosis serves as a common endpoint for stressors ranging from ischaemia to oxidative stress and toxic insults, contributing significantly to morbidity and mortality.

## 2 BLUEPRINT MAPS OF HUMAN AGING

Aging is a complex phenomenon marked by declining cellular function, increased vulnerability to stress, tissue degeneration and a heightened risk of chronic diseases. Over the years, researchers have attempted to distill this complexity into core features, most prominently through typologies such as the ‘hallmarks of aging’ (López‐Otín et al., 2023). These hallmarks, such as genomic instability, oxidative stress and telomere shortening, amongst others, are useful guideposts, but they remain incomplete as far as a framework to understand aging goes. It is still uncertain whether the various hallmarks of aging represent primary causes, downstream effects or merely symptoms of the aging process.

The recently proposed Blueprint Theory (Kern et al., 2024) offers an overarching framework. Rather than cataloguing markers, it seeks to identify core ‘pathological principles’ ‐ the underlying rules that drive biological breakdown. The operation of these principles (i.e., visualizing how those principles play out) is mapped through ‘Blueprint Maps’, which trace how specific gene‐ and molecule‐level changes cascade into pathological cellular and tissue processes such as hyperplasia, transdifferentiation, senescence and fibrosis. In doing so, the Blueprint integrates and contextualizes the hallmarks of aging, reframing the understanding of age‐related disease. One central principle introduced by the Blueprint Theory is the concept of ‘pathological pathways’ or ‘patho‐pathways’. These are normal biological pathways that become harmful when activated (i.e., triggered) in the wrong context (e.g., time, place and/or intensity).

The utility of Blueprint Maps lies in identifying key nodes within complex patho‐pathways ‐ and their interactions with one another ‐ that drive the most detrimental changes in aging and related diseases. This approach parallels the economic principle of factor modelling, which seeks to pinpoint the factors, or ‘nodes,’ driving the largest system‐level shifts. Targeting these nodes is expected to yield the greatest therapeutic benefit.

Within this framework, Blueprint Maps highlight necrosis, i.e. unregulated cell death, as a key pathological process, i.e., a key node (Kern et al., 2025, 2024). Understanding necrosis not merely as an observable cellular event, but as a patho‐pathway, clarifies how it drives systemic degeneration across tissues. By situating necrosis within the Blueprint, its role in aging becomes both more interpretable and more actionable.

Recent findings suggest that aging follows a non‐linear pattern throughout the lifespan, rather than being driven solely by slow, cumulative effects (Argentieri et al., 2025; Shen et al., 2024). Aging can indeed be accelerated in certain contexts, such as space. This supports the idea that specific biological pathways i.e. patho‐pathways, are activated at defined stages, accelerating decline. Among these, necrosis plays a central role, acting as a bridge between stressors (both intrinsic and extrinsic, such as oxidative stress, ischaemia, infections, radiation, toxins and therapeutic side effects) and the onset of deleterious physiological changes, including chronic and persistent inflammation, cell senescence, loss of proteostasis, genomic instability and fibrosis, amongst others (Kern et al., 2025). Put simply, when necrosis accelerates, many of the hallmarks of aging become more pronounced and visible.

## 3 UNDERSTANDING THE ROLE OF NECROSIS IN CHRONIC DISEASE AND AGING THROUGH BLUEPRINT MAPS

Necrosis, though traditionally viewed as chaotic cell death, can be understood to arise primarily from a defined patho‐pathway, initiated when membrane integrity fails and ion gradient homeostasis is disrupted. This disruption leads to a surge in cytosolic calcium levels normally kept several orders of magnitude lower (Fleckenstein et al., 1974). Calcium is a crucial signalling molecule that regulates diverse cellular pathways, but when the gradient collapses the resulting uncontrolled influx of ions triggers a complex calcium‐dependent patho‐pathway that drives necrosis. This includes the hyperactivation of calpain proteases leading to the destruction of internal cellular compartments such as lysosomes (Bonventre, 1996; Fleckenstein et al., 1974; Yamashima et al., 1996). This, in turn, causes the uncontrolled release of hazardous contents into the cytosol, including cathepsins, proteases, oxidative agents, hydrolases, lipases, nucleases, glycosidases and phosphatases, ultimately digesting the cell from the inside out (Yamashima & Oikawa, 2009), which gives necrosis its characteristic eosinophilic, glassy and vacuolated appearance (Majno et al., 1960; Ruffolo, 1964). When individual cells rupture following necrosis, these hazardous contents spill into the surrounding tissue, amplifying damage through the activation of further destructive necrosis patho‐pathways, and hence necrosis, in neighbouring cells. `This extends the reach of the original necrotic event. In the context of aging, this clear mechanistic chain ‐ starting with calcium collapse to systemic tissue damage ‐ makes necrosis a uniquely traceable driver of chronic inflammation, senescence and fibrosis, thereby accelerating biological decline.

**FIGURE 1:**
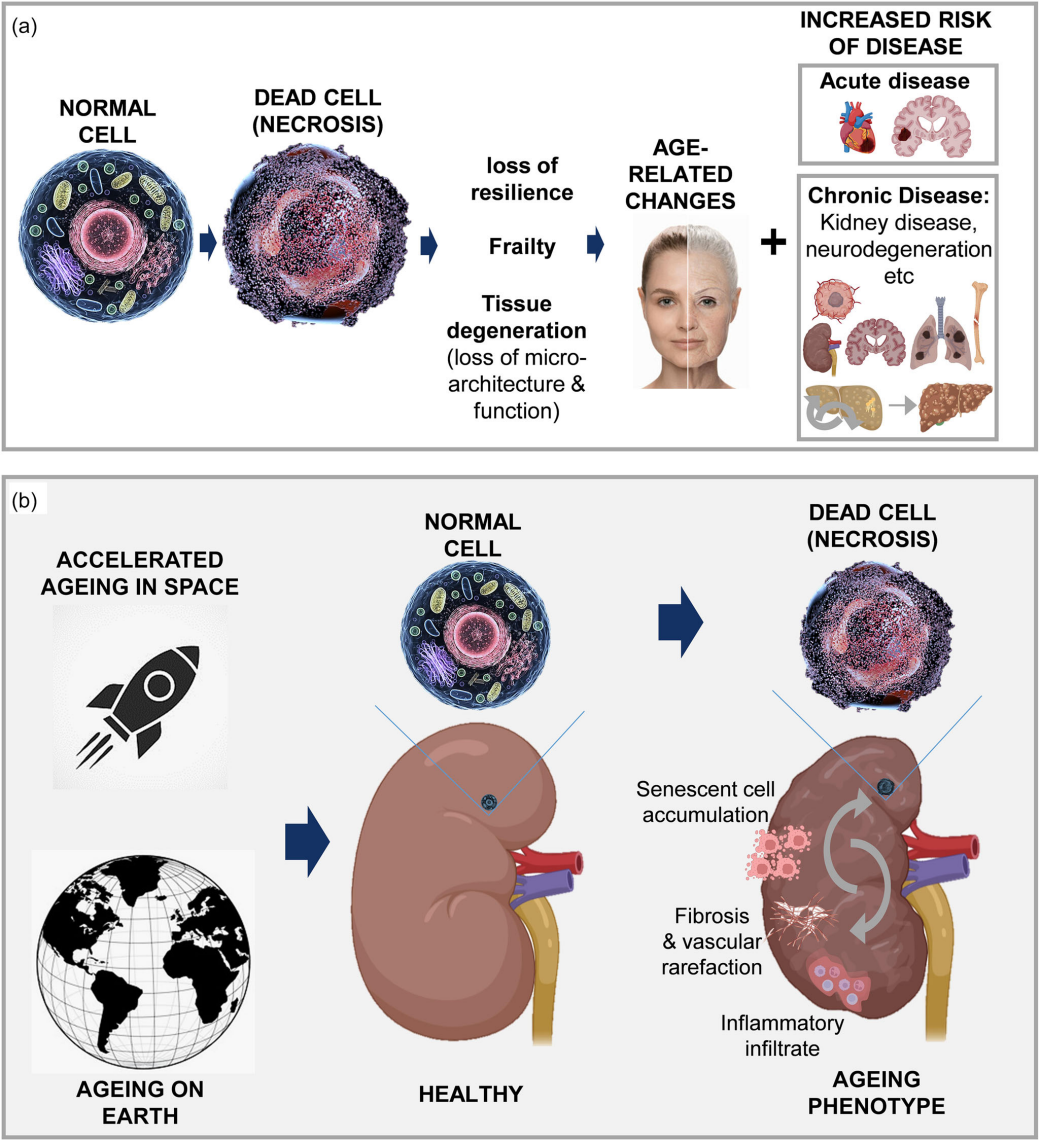
Overview of changes in aging and age‐related conditions on Earth and accelerated phenotype variants in space. (a) Multiple age‐related changes lie upstream of cell necrosis. This is owing to its central role in frailty, loss of resilience with age and tissue degeneration, in addition to positive feedback loops of loss of tissue micro‐architecture and function via induction of direct damage and by association with a poor wound‐healing response, including accumulation of senescent cells, inflammatory infiltrate and fibrosis. (b) Example of mechanistic‐level changes occurring in the kidney that are associated with kidney disease, in addition to some changes observed in space‐associated stress (Ferenbach & Bonventre, 2015; Siew et al., 2024). General changes include cell necrosis‐associated damage and indirect damage via cell senescence, vascular rarefaction, presence of a chronic inflammatory infiltrate, glomerulosclerosis and fibrosis that, in turn, cause loss of kidney structure and function and accelerated kidney aging.

More specifically, necrosis contributes to aging and chronic diseases through three principal mechanisms: (1) in response to severe stress, necrosis causes uncontrolled cell death that directly breaks down tissue architecture and function, i.e., overt tissue degeneration; (2) even when stress is sublethal and does not result in full necrotic cell death, low‐level activation of the necrosis patho‐pathway still disrupts cellular homeostasis. This includes driving genomic instability, loss of proteostasis, telomere shortening, mitochondrial dysfunction, increased oxidative–inflammatory–nitrosative stress and epigenetic changes. Such changes increase frailty and accelerate the onset of chronic diseases via several diverse mechanisms. For example, necrosis‐induced molecular damage can be picked up by programmed cell death pathways like apoptosis, leading to heightened levels of programmed cell death and consequential tissue structural and functional loss, induction of senescent cell states and accumulation, and increasing risk of mutagenesis (Kern et al., 2025); and (3) necrosis initiates maladaptive tissue repair patho‐pathways. Following necrosis, the release of damage‐associated molecular patterns (DAMPs) and harmful cytosolic contents, such as proteases and oxidative agents, provokes chronic immune activation by triggering a patho‐pathway immune response. This process also promotes aberrant senescent cell accumulation and fibrosis as subsequent patho‐pathways, further disrupting tissue homeostasis and increasing the susceptibility of tissues to additional damage (Ferenbach & Bonventre, 2015; Kern et al., 2025, 2024). These downstream effects create vicious feedback loops that amplify damage and accelerate biological degeneration. It is these feedback loops of patho‐pathways that also prevent tissue regeneration (Figure 1a).

Together, these three mechanisms illustrate how necrosis acts as a key interface between stressors (i.e., the exposome) and the biological aging process, with its effects traceable at a mechanistic level (Kern et al., 2024; Woods et al., 2025).

## 4 SPACE EXPLORATION AS A NATURAL LABORATORY

We are moving toward a new era of human spaceflight, driven by sustainable exploration, space tourism and long‐duration missions beyond low Earth orbit. Programmes like Artemis, in collaboration with the National Aeronautics and Space Administration (NASA) and the European Space Agency (ESA), aim to establish a lunar outpost as a stepping stone to a crewed Mars mission by the late 2040s. While technological advancements progress quickly, human biology could be the ultimate barrier as we transition from a terrestrial to a space‐faring species. Ongoing experiments will be crucial for overcoming this hurdle, such as those on the recent Fram2 mission by SpaceX that achieved the first human spaceflight over Earth's polar regions and those ongoing on the International Space Station (ISS), including work on diagnostics, health changes and potential therapeutic strategies. Examples include capturing the first human X‐ray images in space, the effect of blood‐flow restriction on muscle and bone health, and effects on cell culture and cell survival and proliferation in space.

The conditions of space travel i.e. microgravity, cosmic radiation, physical deconditioning (a decline in health resulting from physical inactivity, as commonly observed during extended hospitalizations, stays in intensive care units or among individuals with sedentary lifestyles) and prolonged isolation, subject the human body to significant stress. Such stresses accelerate aging‐related processes such as bone density loss, muscle atrophy, neuronal dysfunction (including optic disc swelling, choroidal folds, posterior globe flattening and optic nerve sheath distention) and immune cell death and dysfunction (Cohen‐Jonathan et al., 1999; Lv et al., 2023; Mader et al., 2011; Siew et al., 2024).

Astronauts, who experience a “fast‐forwarded” version of aging and organ damage, offer a unique model for studying cellular stress and resilience. Necrosis is one such accelerated degenerative mechanism.

The mechanisms in space that initiate accelerated aging phenotypes include the combined effects of haemodynamic stress (e.g., posterior cerebral hyperperfusion, which promotes minor blood–brain barrier disruption), ischaemic stress, physical deconditioning and systemic oxidative stress, all of which contribute to and are exacerbated by chronic inflammatory pathways (Bailey et al., 2020; Montesinos et al., 2021; Riley et al., 1996). These factors culminate in disrupted redox homeostasis, triggering necrosis through the loss of cell membrane integrity. Cells particularly sensitive to necrosis in space include kidney tubular epithelial cells, neuronal white matter cells, innate immune cells and retinal cells (Ding et al., 2011; Li et al., 2013; Panesar et al., 2020; Philpott et al., 1978; Riley et al., 1996).

## 5 NECROSIS: A CHALLENGE FOR HUMAN HEALTH IN SPACE AND ON EARTH

In space and on Earth, necrosis remains an unresolved therapeutic challenge. Despite its central role in kidney disease, neurodegeneration, cardiovascular decline and wider medical challenges, such as deconditioning, no pharmacological intervention has successfully blocked necrosis in the clinic. Broad strategies aimed at targeting upstream stressors, such as anti‐inflammatories and antioxidant therapies, have resulted in multiple failed clinical trials for chronic diseases associated with necrosis, including kidney disease and cardiovascular disease (Bulger et al., 2020; Dvirnik et al., 2018; Lamy et al., 2024; Pickkers et al., 2018, 2022; van Till et al., 2023). Attempts to directly block necrosis at the cell level have also failed to date. While, inhibiting proteases such as calpains and cathepsins has shown some protective effects in vitro, these strategies are limited because these enzymes act downstream in necrotic cascades, intervening only after significant cellular damage has already occurred (Fleckenstein et al., 1974). The most promising approach to date is to block the upstream trigger of necrosis ‐ the loss of cytosolic calcium ion homeostasis (Fleckenstein et al., 1974). However, this approach has shown limited success, and the reasons remain unresolved. Early studies using membrane calcium channel blockers, such as nifedipine, verapamil and other dihydropyridines, initially showed promise in animal models of ischaemic stress. Yet, further research revealed that the observed benefits were primarily attributable to the vasodilatory effects of these calcium channel blockers rather than direct inhibition of necrosis. Consequently, the effects of these blockers are not conserved across stressors and are of limited relevance to the clinic. This is further reflected in numerous failed clinical trials with these drugs (Dhir et al., 2020; Franke et al., 1996; Jensen, 2002; Oczkowski et al., 1989; Wahlgren, 1997; Wahlgren et al., 1994).

This is where space becomes uniquely valuable. While the destructive patho‐pathway cascades initiated by necrosis—such as those leading to aging, chronic disease and frailty—typically unfold slowly and diffusely on Earth, the environment of space accelerates these pathologies, offering the ability to study them in compressed time frames. By identifying and targeting the molecular patho‐pathways associated with necrosis in this setting, researchers may uncover entirely new strategies to intervene early in disease processes, potentially breaking damage feedback loops and promoting tissue repair.

Such interventions could offer broad therapeutic potential. Since necrosis is a conserved biological mechanism underlying many chronic diseases, a successful treatment could simultaneously improve outcomes across multiple conditions and even help to prevent disease onset altogether.

Beyond natural aging, the benefits might also extend to factors that induce accelerated physiological decline in younger individuals on Earth. One example is the rapid aging and disease onset that can follow physical deconditioning. Individuals with sedentary lifestyles or those who are bedbound, even for a few days, are vulnerable. This is particularly true for patients undergoing prolonged hospitalizations, extended stays in intensive care units or lengthy surgical recoveries. These individuals often experience a range of accelerated aging‐related conditions similar to those observed in astronauts subjected to prolonged inactivity in space. Common manifestations include sarcopenia and osteoporosis, many of which can persist even after discharge (Chen et al., 2011; Kehler et al., 2019; Wann‐Hansson et al., 2008; Welch et al., 2018).

In short, space not only provides the conditions to accelerate discovery, but it may also hold the key to unlocking new classes of treatments for aging and chronic disease on Earth.

## 6 COULD KIDNEYS LEAD THE WAY?

Moving to specific diseases, kidney disease in particular stands out as a compelling case. As our prior work shows, the kidney is especially vulnerable to damage in space, making it both a sentinel organ for studying necrosis and a high‐impact target for intervention (Kern & Siew, 2026; Siew et al., 2024).

Kidney disease is one of the most pressing health challenges. It ranks as the 9th leading cause of death (WHO, 2024) and the 3rd fastest growing cause of death globally (predicted to be the 5th highest cause of years of life lost by 2040) (Francis et al., 2024). The kidney's vulnerability stems from its high metabolic demands, intricate microvascular network, and sensitivity to necrosis due to its various blood pressure, fluid and electrolyte regulatory functions; the kidneys take 20%–25% of the cardiac output while only constituting of around 1% of total body mass (Moe et al., 2009).

On Earth, stressors such as ischaemia, oxidative damage, inflammation and nephrotoxic drugs (e.g., chemotherapy drugs) drive necrosis in tubular epithelial cells (termed acute tubular necrosis or ATN), leading to loss of microarchitecture and functional decline. If the stress is severe, ATN results in acute kidney injury (AKI), marked by impaired organ function, including disrupted water and electrolyte homeostasis and reduced excretion of metabolic waste products (Bonventre & Yang, 2011; Ferenbach & Bonventre, 2015).

AKI, in turn, is closely linked to chronic kidney disease (CKD), with severe or untreated AKI potentially progressing to CKD (Ferenbach & Bonventre, 2015). In addition to direct tissue damage from necrosis, as described earlier, secondary injury in the kidney occurs through necrosis propagation causing damage patho‐pathway cascades, including via the release of DAMPs and harmful cellular contents, like digestive proteases. This promotes maladaptive repair, leading to persistent structural and functional abnormalities in the kidney. Patho‐pathways of maladaptive repair from necrosis entail senescent cell accumulation, fibrosis, vascular rarefaction, tubular loss, glomerulosclerosis and chronic inflammation, all contributing to CKD (Basile et al., 2001; Grgic et al., 2012; Yang et al., 2010). Lower‐grade initiation of the necrosis patho‐pathway and associated cellular damage can also damage renal tubular epithelial cells, arresting them in the G2/M phase and inducing a profibrotic phenotype that affects surrounding cells, pericytes and the immune system (Yang et al., 2010). Additionally, myofibroblasts, likely derived from renal pericytes, proliferate and contribute to fibrosis in the injured kidney (Bonventre & Yang, 2011).

CKD resembles an ‘accelerated’ form of kidney aging, sharing many features of natural kidney aging (Ferenbach & Bonventre, 2015). This state, including immune dysfunction and genetic instability in necrotic regions, also increases the risk of renal carcinoma (Chow et al., 2010).

These mechanisms make kidney disease a relevant model from an economic and disease burden perspective, as well as an ideal model for studying necrosis in the context of aging ‐ particularly in an organ that exhibits accelerated aging and decline compared to others. The kidney mirrors processes that occur at slower rates across the rest of physiology, such as inflammaging, cell senescence and fibrosis, alongside loss of resilience to stress and regenerative capacity, frailty and tissue degeneration. It is also because of this that maintenance of kidney health alongside risk of accelerated aging from microgravity and radiation will be particularly challenging and a key limiting factor to long‐duration space missions (Siew et al., 2024).

Thus, the kidney is a very relevant organ to study for both Earth‐based and astronaut‐related health (Figure 1b). Targeting necrosis in the kidney could disrupt crucial damage feedback loops, offering therapeutic pathways not only for pushing the frontiers of human space exploration but also to address chronic disease and aging more broadly on Earth.

## 7 FROM EVOLUTION TO INTERVENTION: WHY SPACE‐AGE MEDICINE NEEDS SYSTEM‐LEVEL PRECISION

For context, from an evolutionary perspective, cellular and tissue‐level changes associated with functional plasticity (such as inflammation, senescence, fibrosis and cell remodelling) come with inherent trade‐offs. While the pathways that govern these processes are essential for critical functions like wound healing, they can also be triggered aberrantly as patho‐pathways to become maladaptive in the context of aging, contributing to chronic disease and degeneration via processes such as inflammaging (Ferenbach & Bonventre, 2015; Kern et al., 2024). This is accelerated in space. The core issue is that biological functions cannot be optimized for all circumstances, as evolutionary constraints inherently force trade‐offs (Kern et al., 2024; Williams, 1957).

Biology functions as a complex system of trade‐offs, shaped by a network of evolutionary constraints that optimize survival and reproduction across different environments (Kern et al., 2024). Disease mechanisms, such as patho‐pathways, emerge when this delicate balance is disrupted by intrinsic or extrinsic factors, leading to unintended trade‐offs. A key challenge in any intervention, particularly those targeting aging or chronic diseases, is the risk of introducing new trade‐off costs. For example, rapamycin can protect against bone osteoporosis by suppressing bone remodelling, but it also hinders fracture healing (Holstein et al., 2008; Shen et al., 2018). Similarly, Glucagon‐Like Peptide‐1 (GLP‐1) receptor agonists can reduce body fat by their central effect on the hypothalamus and improve health, however, if weight loss is too rapid, the individual's facial features appear to rapidly age as the facial fat and muscle volume decrease in a pathological manner (i.e., lipodystrophy and sarcopenia risk). These include a sunken appearance in the temples and cheeks, more prominent nasolabial folds and drooping of the skin around the periorbis and jaw line. A phenomenon often called ‘Wegovy face’ or ‘Ozempic face’ (Humphrey & Lawrence, 2023). General sarcopenia and lipodystrophy are also risks, alongside knock‐on consequences such as rapid fat loss stressing the retina to induce macular degeneration (Ren et al., 2025; Shor et al., 2025). As other examples, Bisphosphonates used to treat hypercalcaemia (high blood calcium) associated with bone‐resorption conditions such as in cancer and osteoporosis, can cause necrosis of the bone due to altered wound healing and delayed epithelial closure of a mucosal opening in the mouth (Rizzoli et al., 2008). Glucocorticoids prescribed to reduce inflammation in giant cell arteritis can lead to avascular necrosis of the hip by impairing blood vessel function, including inhibiting angiogenesis (Chan & Mok, 2012).

In space, where stressors like radiation and microgravity disrupt the system of finely tuned biological trade‐offs and rapidly destabilize biology, the need for interventions that do not further destabilize by over‐correction in the system is paramount. The aim is to reduce unintended biological trade‐offs, while maintaining a Treat‐to‐Target (T2T) approach (i.e. not merely addressing symptoms, but proactively guiding and adjusting treatment to achieve and sustain defined health outcomes (Dejaco et al., 2024; van Vollenhoven, 2019)). This is particularly important when applying a prevention‐oriented T2T strategy to mitigate the early onset of degeneration.

Achieving this requires a detailedand systems‐level understanding of the complex network of biological trade‐offs that govern aging and disease. Blueprint Maps provide a way to trace how the various patho‐pathways interact and drive degeneration. This in turn allows the identification of key intervention points ‐ specific nodes in the network where interventions can have maximum effect with minimal predicted unintended consequences (Kern et al., 2024). The necrosis patho‐pathway is a prime example of such a node; it is a non‐adaptive, non‐genetically programmed, purely destructive process that offers no physiological benefit, making its inhibition a low‐risk, high‐reward strategy.

Identifying such nodes for intervention is only part of the challenge. Advances in theory, such as the Blueprint Theory, together with increasingly sophisticated biomarkers and mechanistic tools (e.g., omics techniques), are improving our ability to map the pathways that drive degeneration. However, many of these pathways serve important physiological functions and become harmful only when activated in the wrong time, in the wrong place, or to the wrong extent. So how do we safely intervene in these more complex, risk‐prone nodes, where therapeutic gains may come at the cost of triggering unwanted trade‐offs? And how do we take a more targeted approach, accounting for underling variations in the drivers of even the same disease across individuals, i.e., work towards a precision medicine approach?

## 8 THE ‘CHAMELEON EFFECT’: A PHARMACOLOGICAL STRATEGY FOR TARGETING PATHO‐PATHWAYS IN SPACE BIOLOGY

Once a key pathological node is identified, the next challenge is identifying which therapeutic modalities are best suited to intervene without introducing additional trade‐offs and further destabilizing the system. In the quest to combat age‐related processes both on Earth and in space, pharmacological approaches could offer distinct advantages over gene editing: enhanced plasticity of strategy, and thus potentially greater capacity for navigating biological trade‐offs. As explained above, aging and chronic diseases often stem from the dysregulation of otherwise adaptive cellular activities i.e. processes that go awry in the wrong context or at the wrong time, and where capacity for cellular plasticity is a double‐edged sword. These misfires, known as patho‐pathways, act like faulty nodes in an electrical grid, driving damage. Because drugs can often modulate these pathways transiently and reversibly, they may allow cellular responses to be adjusted without the durable alterations associated with many current gene‐editing and ‐therapy approaches; that is, drugs can act like smart switches as they temporarily and reversibly adjust malfunctioning nodes without permanently rewiring the system.

We term this capacity for reversible and context‐dependent modulation the ‘chameleon effect’: the ability to adapt therapeutic responses to changing biological circumstances without inducing irreversible change. More broadly, the chameleon effect reflects a therapeutic principle: because many age‐related pathologies arise from otherwise adaptive biological processes activated inappropriately, effective interventions must themselves be capable of adapting to biological context.

Gene editing and therapy, while powerful, can carry risks associated with durable or irreversible genomic changes. Moreover, its applicability is limited by the complexity of aging, which involves numerous interacting and dynamic pathways. It is precisely this complexity ‐ marked by feedback loops, pleiotropy and context‐specific pathway activation ‐ that makes flexible modulation so valuable. Returning to the earlier example of rapamycin, its benefits appear to depend not on permanent mechanistic Target of Rapamycin (mTOR) suppression, but on ad hoc, temporally calibrated dosing. Thus, a key question moving forwards is whether emerging gene‐editing and therapy technologies will evolve to offer the same level of temporal and contextual precision as pharmacology. Until such technologies mature, drugs could remain our most versatile tools for navigating biological trade‐offs.

To be clear, the chameleon effect does not argue that one therapeutic modality is universally superior. Rather it argues that the optimal modality depends on the biological characteristics of the target. For the dynamic and context‐dependent patho‐pathways that are arguably at the heart of much of the age‐related degeneration, reversible pharmacological modulation may offer distinct advantages. Fully harnessing this ‘chameleon effect’ thus requires a deep understanding of the causal mechanisms that drive pathological change. This includes accounting for environmental (exposomic) triggers and mapping the constraints within which biology operates. The creation of ‘Blueprint Maps’ offers a structured approach to identify precise leverage points for intervention with minimal unintended consequences (Kern et al., 2024).

## 9 CONCLUSION

Rather than distracting from challenges on Earth, space exploration offers a rare opportunity to study biological processes in ways that are difficult to replicate terrestrially. The extreme environment of space functions as a biological stress test, accelerating physiological decline and exposing vulnerabilities in human biology. In this accelerated model of decline, we gain unique insights into aging and chronic diseases.

These findings have practical implications. They point us towards high‐impact intervention points ‐ patho‐pathways that, when better understood, could shift the trajectory of health and disease. Necrosis is one such mechanism: a non‐programmed form of cell death triggered by overwhelming stress It plays a central role in many of the physiological breakdowns observed both in space and on Earth ‐ from tissue degeneration and inflammation to chronic disease. Understanding how and when patho‐pathways like necrosis occur, offers us a chance to intervene before irreversible damage sets in, and to preserve physiological resilience before it is lost.

Crucially, the biological changes observed during spaceflight are not static; they emerge from a highly dynamic interplay between stress, adaptation and degeneration. This highlights not only the importance of identifying pathological mechanisms, but also the need for interventions that can respond flexibly to changing biological contexts.

Targeting these systemic patho‐pathways requires nuance, what we term the ‘chameleon effect’: the ability to shift cellular behaviour flexibly in response to context, turning down harmful patho‐pathways while preserving beneficial ones. This flexibility allows intervention while reducing the risk of introducing additional biological trade‐offs.

As we prepare for the next frontier of human exploration, these lessons from space biology could have far‐reaching impact ‐ not only helping us to survive beyond Earth, but also transforming the way we understand and treat aging, disease and resilience here at home.

## AUTHOR CONTRIBUTIONS

All authors contributed equally to the manuscript. Carina Kern conceptualized and wrote the first draft, after which Tina Woods and Keith Siew contributed equally to the development and revision of the manuscript. All authors approved the final version of the manuscript and agree to be accountable for all aspects of the work in ensuring that questions related to the accuracy or integrity of any part of the work are appropriately investigated and resolved. All persons designated as authors qualify for authorship, and all those who qualify for authorship are listed.

## CONFLICT OF INTEREST

Tina Woods is CEO of Collider Health and trustee of the British Society for Research on Aging.

## FUNDING INFORMATION

This work did not receive any funding from public, commercial, or not‐for‐profit organizations.

## GENERATIVE AI STATEMENT

Generative AI assistance (OpenAI ChatGPT) was used to check and improve the grammar, language, readability, and clarity of the manuscript. All scientific content, interpretations, and conclusions were developed and verified by the authors, who take full responsibility for the content of the publication. Image creation: Figures were created partially under license in BioRender and partially using images under license from iStock.com.
